# Tacrolimus Aggravated Tube Feeding Syndrome with Acute Renal Failure in a Pediatric Liver Transplant Recipient

**DOI:** 10.1155/2017/7345680

**Published:** 2017-08-20

**Authors:** R. Kula, M. Melter, J. Kunkel, C. Dörfler, S. Alikadic, B. Knoppke, R. Zant

**Affiliations:** KUNO University Children's Hospital, Regensburg, Germany

## Abstract

Acute renal failure can be caused by calcineurin inhibitors (CNIs), due to arteriolopathy and altered tubular function. Within this context, we present the case of a 14-month-old liver transplant recipient who suffered an acute polyuric renal failure during a short episode of hypercaloric feeding. In our case, CNI-induced distal RTA led to nephrocalcinosis and therefore to secondary nephrogenic diabetes insipidus. The diet with high renal solute load consequently resulted in an acute polyuric renal failure with severe hypernatremic dehydration. In conclusion, a hypercaloric diet in children with potentially impaired renal function due to therapy with CNIs requires precise calculation of the potential renal solute load and the associated fluid requirements.

## 1. Introduction

Tacrolimus and cyclosporine are widely used calcineurin inhibitors (CNIs) with a narrow therapeutic window and multiple drug-drug interactions [1]. They have the potency to induce renal failure due to acute arteriolopathy. Moreover, CNIs lead to altered tubular function, resulting in an impaired renal concentrating ability [2].

Within this context, we report the case of a pediatric liver transplant recipient who suffered acute polyuric renal failure leading to severe dehydration during a diet with high renal solute load.

Informed consent was obtained from the parents for publication of this report.

## 2. Case Presentation

A 14-month-old girl with a body weight of 7.7 kg was admitted to our pediatric intensive care unit (PICU) in acute polyuric renal failure including severe hypernatremia, hyperchloremia, hyperkalemia, hyperuricemia, and metabolic acidosis. Clinically she was irritable and appeared only mildly dehydrated. Her heart rate on admission was 150 beats per minute with an arterial blood pressure of 83/43 (63) mmHg. Renal ultrasound on admission revealed bilateral nephrocalcinosis type IIa. Her laboratory findings on admission are summarized in Table 1. The girl was treated with tacrolimus after liver transplantation 7 months prior to admission for extrahepatic biliary atresia. Renal ultrasound before liver transplantation showed a normal finding. She was discharged home from a former hospital stay 8 days prior to this admission with the recommendation for 800 ml Nutrini Energy MultiFibre® (Table 2) a day in addition to complementary feeding. Against medical advice she drank no additional water. Therefore, her calculated water deficit over the last 8 days was 23.6% of her body weight (Table 3). In PICU the high protein diet was interrupted and the girl was intravenously rehydrated. The average diuresis in the first 12 hours after admission was 8.1 ml/kg/hour. Additionally, bicarbonate was administered and the antihypertensive therapy with enalapril was paused for 2 days. Under these therapeutic measures the renal function recovered and the serum electrolytes returned to normal values. After reinstating feeding with Nutrini MultiFibre plus oral bicarbonate instead of Nutrini Energy MultiFibre the child was transferred to the normal ward. On day 9 after admission she was discharged home. At the time of discharge the patient's serum urea and creatinine values were within the normal range, her glomerular filtration rate assessed by serum cystatin C was 76 ml/min/1.73 sqm. In the later course immunosuppression was changed from tacrolimus monotherapy to low-dose tacrolimus plus mycophenolate mofetil.

## 3. Discussion

The etiology of calcineurin inhibitor induced nephrotoxicity has not been clearly established yet. It is thought to be multifactorial, resulting from a combination of an increase in vasoconstrictive factors (endothelin and thromboxane), activation of the renin-angiotensin-aldosterone system, reduction of vasodilator factors (nitric oxide and prostacyclin), and formation of free radicals. CNIs also lead to tubular functional alterations and ion homeostasis disturbances like hyperkalemia, hypomagnesemia and magnesium wasting, distal tubular acidosis, and hyperuricemia. Some of the effects of CNIs on tubular function can be explained by reduced expression of the Na^+^-K^+^-2Cl^−^-cotransporter (NKCC2) on the apical membrane of tubular epithelial cells. Decreased expression of NKCC2 would lead to polyuria, nephrocalcinosis, magnesium wasting, and hyperreninemic hyperaldosteronism [5]. As mentioned above, distal RTA is one of several features of nephrotoxicity induced by tacrolimus treatment [2]. Patients with distal RTA have a metabolic acidosis with an inability to acidify the urine appropriately. This type of RTA is caused by impaired distal acidification and is characterized by the inability to lower urinary pH maximally (<5.5) under the stimulus of systemic acidemia [6]. Hypercalciuria and nephrocalcinosis are typically present. The mainstay of therapy in all forms of RTA is bicarbonate replacement. The base requirement for distal RTAs is generally in the range of 2–4 mEq/kg/24 hours. Patients with distal RTA should be monitored for the development of hypercalciuria [7]. The defining characteristic of nephrocalcinosis is generalized calcium deposition in the kidney. Nephrocalcinosis can involve the renal medulla or, much less often, the cortex. The most common cause of nephrocalcinosis is increased urinary calcium excretion with or without hypercalcemia. Distal RTA is the most common cause of nephrocalcinosis (particularly in children) due to hypercalciuria without hypercalcemia. The reported prevalence of nephrocalcinosis in patients with distal RTA ranges from 60 to 80 percent. However, it is clinically difficult to differentiate since nephrocalcinosis itself frequently causes defects in distal acidification. Nocturia, polyuria, and polydipsia due to an impaired urinary concentrating ability (nephrogenic diabetes insipidus) may occur in patients with hypercalcemia or medullary nephrocalcinosis of any cause [8]. Recent data support the hypothesis that in vivo external calcium, through activation of calcium-sensing receptors, modulates the expression/trafficking of aquaporin 2 (AQP2). Short-term control of water permeability occurs via vesicular trafficking of AQP2 and long-term control through changes in the abundance of AQP2 and AQP3 water channels. Defective AQP2 trafficking causes nephrogenic diabetes insipidus, a condition characterized by the kidney's inability to produce concentrated urine because of the insensitivity of the distal nephron to vasopressin [9]. The underlying cause of nephrocalcinosis should be determined and treated if possible. No specific treatment has been shown to prevent progression of nephrocalcinosis. Increased fluid intake may be beneficial for all patients with nephrocalcinosis. Among patients with hypercalciuria, urinary calcium excretion may be reduced by dietary modifications that include restriction of animal protein and sodium intake [10]. Infant formulas have a lower protein content and therefore a lower renal solute load compared to products designed for patients aged over 1 year. Children with increased metabolic needs (e.g., after liver transplantation) and/or decreased fluid tolerance may not be able to consume an adequate volume of standard formulas to promote growth. In this instance, a more concentrated formula may be needed. However, a protein intake which accounts for more than 16% of calories could contribute to azotemia and negative water balance if associated fluid intakes are low. In these cases the caloric density can be increased by utilizing modular additives of carbohydrate or fat because both do not increase the renal solute load. The potential renal solute load does not need to be calculated routinely, but it is important with patients who have medical problems [11]. If healthy infants consume a predominantly liquid diet ad libitum, their ability to excrete unneeded solutes and to maintain water balance is more than adequate [3]. But the renal solute load and fluid balance should be closely monitored if children have a low fluid intake, if they are receiving calorically dense feedings, or if they have increased extrarenal fluid losses and/or an impaired renal concentrating ability. In these children additional water may be required [12].

In summary, renal dysfunction in pediatric liver transplant recipients treated with CNIs like tacrolimus is well known and well documented in the existing literature [13]. The overall prevalence of chronic kidney disease in this population ranges up to 32% [14]. Renal failure due to nephrocalcinosis in pediatric liver transplantation is usually described in the context of hyperoxaluria; however, information on tacrolimus induced acute renal failure associated with nephrocalcinosis is scarce and to the best of our knowledge this is the first describing report [15]. Previously published reports include early graft failure in an adult kidney transplanted recipient with severe renal calcifications under tacrolimus treatment [16]. A likely pathophysiologic scenario in our case may have been that tacrolimus induced distal RTA led to nephrocalcinosis and therefore to secondary nephrogenic diabetes insipidus. Insufficient water intake in a diet with high renal solute load and further renal impairment due to enalapril treatment consequently resulted in an acute polyuric renal failure with severe hypernatremic dehydration.

## 4. Conclusion

A hypercaloric diet in children with potentially impaired renal function due to therapy with CNIs requires precise determination of the potential renal solute load and the associated fluid requirements. Pediatric liver transplant recipients treated with CNIs should be assessed for nephrocalcinosis and associated impaired renal function at regular intervals.

## Figures and Tables

**Table 1:** Laboratory findings on admission to the pediatric intensive care unit.

Laboratory findings on admission	
Serum pH	7.19
Serum bicarbonate	11.8 mmol/L
Serum base excess	−15 mmol/L
Serum sodium	170 mmol/L
Serum potassium	7.4 mmol/L
Serum chloride	143 mmol/L
Serum calcium	1.9 mmol/L
Serum phosphate	2.85 mmol/L
Serum creatinine	1.6 mg/dL
Serum urea	364 mg/dL
Serum osmolality (measured)	425 mmol/kg
Tacrolimus level	7.7 mcg/L
Urine pH	5.6
Urine osmolality	465 mmol/kg
Urine sodium/creatinine ratio	86 (mM based reference: <58)
Urine potassium/creatinine ratio	25 (mM based reference: <68)
Urine phosphate/creatinine ratio	18 (mM based reference: <14)

**Table 2:** Nutrini Energy MultiFibre.

Nutrini Energy MultiFibre	
Energy	1.5 kcal/ml
Protein	40 g/L
Sodium	39,1 mmol/L
Chloride	40.3 mmol/L
Potassium	42.2 mmol/L
Calcium	22.4 mmo/L
Phosphorus	24.2 mmol/L
Nitrogen	6400 mg/L
Osmolarity	315 mosmol/L
Potential renal solute load	374 mosmol/L

**Table 3:** The patient's calculated water deficit over the last 8 days prior to admission to the pediatric intensive care unit.

*Equations*
Water intake = food intake × water content
Water requirement = (food intake × potential renal solute load/age dependent estimated maximal renal concentration capacity) + (insensible water losses × patient's body surface area)
Water deficit = water intake − water requirement

*Case specific calculations*
Water intake per day = 0.8 L/day × 0.77 L/L = 0.616 L/day
Water requirement per day = (0.8 L/day × 374 mOsmol/L/465 mOsmol/L) + (0.5 L/day/sqm × 0.4 sqm) = 0.843 L/day
Water deficit per day = 0.616 L/day − 0.843 L/day = −0.227 L/day
Water deficit within 8 days = 1.820 L
Percent of body weight = 1.820 L/7.7 kg*⁡*~23.6%

*Potential renal solute load (PRSL)* refers to solutes of dietary origin that would need to be excreted in the urine if none were diverted into synthesis of new tissue or lost through nonrenal routes. It is calculated by the following equation: PRSL = nitrogen/28 + sodium + chloride + potassium + phosphorus. The units are in milliosmoles, except for nitrogen, which is total nitrogen in milligrams. Available phosphorus is assumed to be total phosphorus of milk-based formulas and two-thirds of the phosphorus of soy-based formulas. PRSL is expressed as milliosmoles per liter [3]. The renal concentration capacity on admission was 465 mOsmol/L. The insensible fluid losses under normal condition are 0.5 L/sqm/day [4].

## Abbreviations

AQP2: Aquaporin 2
CNIs: Calcineurin inhibitors
NKCC2: Na^+^-K^+^-2Cl^−^-cotransporter
PICU: Pediatric intensive care unit
PRSL: Potential renal solute load
RTA: Renal tubular acidosis.
